# Direct dating of Pleistocene stegodon from Timor Island, East Nusa Tenggara

**DOI:** 10.7717/peerj.1788

**Published:** 2016-03-10

**Authors:** Julien Louys, Gilbert J. Price, Sue O’Connor

**Affiliations:** 1 Department of Archaeology and Natural History, Australian National University, Canberra, Australian Capital Territory, Australia; 2 School of Earth Sciences, The University of Queensland, St Lucia, Queensland, Australia

**Keywords:** Geochronology, Proboscidean, Extinction, Island biogeography

## Abstract

Stegodons are a commonly recovered extinct proboscidean (elephants and allies) from the Pleistocene record of Southeast Asian oceanic islands. Estimates on when stegodons arrived on individual islands and the timings of their extinctions are poorly constrained due to few reported direct geochronological analyses of their remains. Here we report on uranium-series dating of a stegodon tusk recovered from the Ainaro Gravels of Timor. The six dates obtained indicate the local presence of stegodons in Timor at or before 130 ka, significantly pre-dating the earliest evidence of humans on the island. On the basis of current data, we find no evidence for significant environmental changes or the presence of modern humans in the region during that time. Thus, we do not consider either of these factors to have contributed significantly to their extinction. In the absence of these, we propose that their extinction was possibly the result of long-term demographic and genetic declines associated with an isolated island population.

## Introduction

Stegodons (Stegodontidae: Proboscidea) were a widespread and diverse family of proboscideans dating from the late Miocene to the Late Pleistocene of Africa and Asia. Pleistocene species of stegodon were widely dispersed throughout continental and island Southeast Asia (Louys, Curnoe & Tong, 2007), but became globally extinct by the terminal Pleistocene on both the mainland (Turvey et al., 2013) and on islands (Van den Bergh et al., 2008). Ultimate causes for their global extinction are likely multi-faceted, although it’s been proposed that continental and insular forms were adversely affected by environmental changes associated with glacial-interglacial cycles and volcanic eruptions, respectively (Louys, Curnoe & Tong, 2007; Louys, 2008; Van den Bergh et al., 2008). In Southeast Asia, stegodons are remarkable for being one of only a few large-bodied species to be relatively common in the fossil record of both small and large islands (Van den Bergh, 1999). Moreover, the occurrence of stegodons on islands that have never been connected to continents means they were commonly the only large- or medium-sized mammals to inhabit these until the later arrival of anatomically modern humans. Genetic and geographic isolation of stegodon populations resulted in dwarfing, speciation, and extinctions, with these processes playing out on different islands at different times (Van den Bergh, 1999; Van der Geer et al., 2010). They are a charismatic Asian example of the ‘island rule’: the ecological processes proposed to explain the phenomenon by which large-bodied insular animals become smaller than mainland forms, and small-bodied animals become larger (Foster, 1964; Lomolino et al., 2013). Stegodons are thus important for understanding some key biological phenomena including the dispersal abilities of large-bodied mammals and the evolutionary mechanisms of island dwarfing.

**Figure 1:**
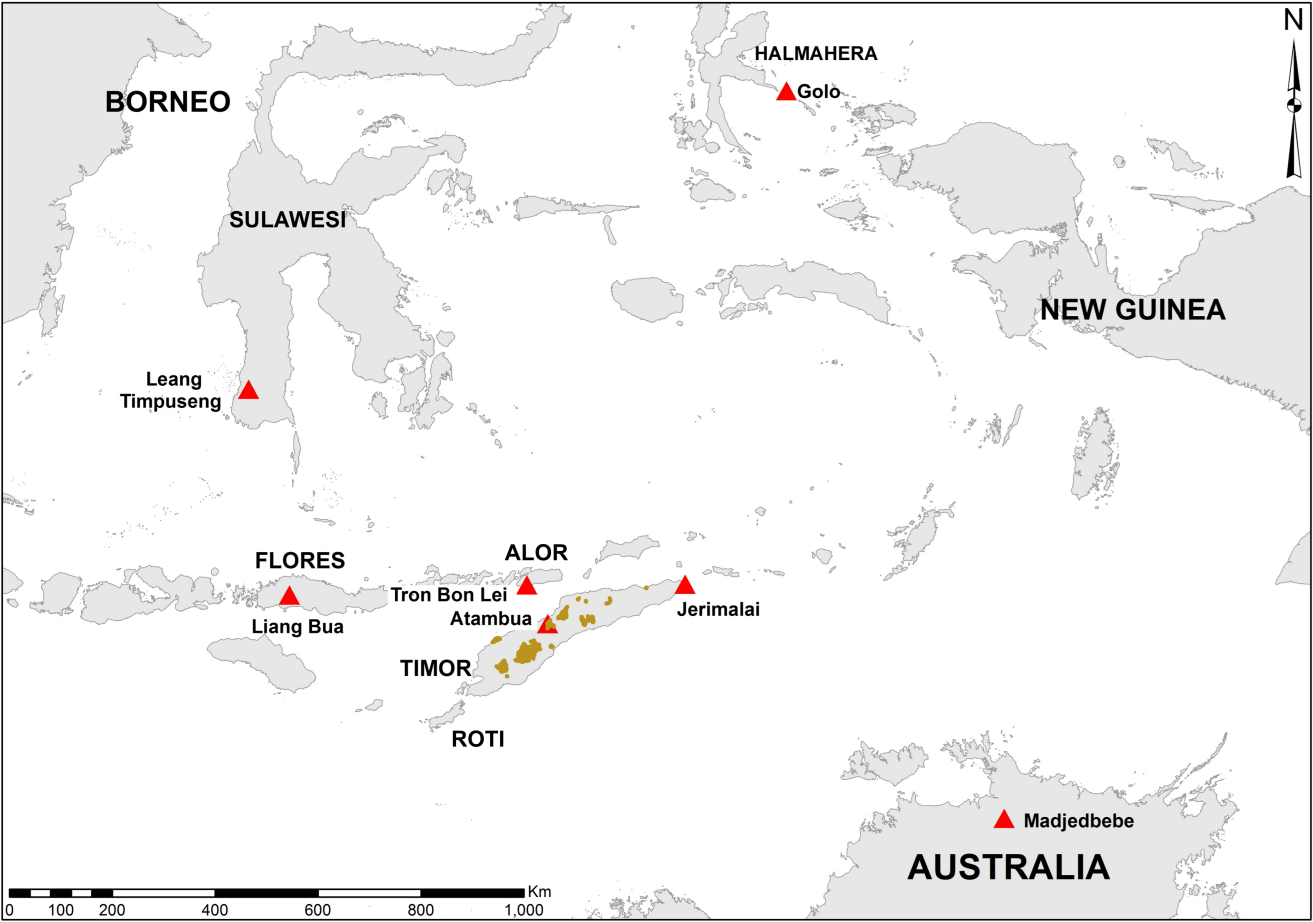
Map of area. Regional map showing the location of the major sites discussed in the text (red triangles). Outcrops of the Ainaro Gravels on Timor are highlighted in brown.

In 1964, the archaeologist Theodore Verhoeven travelled to the island of Timor (Fig. 1) in order to follow up on his earlier success of finding stegodon and stone tools in Flores. His visit was a success, as he found both stegodon molar fragments and stone tools in the vicinity of the village of Atambua, in what is now West Timor, Indonesia (Verhoeven, 1964). Verhoeven’s (1964) brief report provided sufficient details of geographic location and stratigraphic provenance for the stegodon fossils to allow subsequent researchers to relocate, and in some cases re-excavate, the fossil-bearing deposits. The first documented researcher to do so was Hooijer, who visited Flores, Timor, and Sulawesi in 1970 (Van den Bergh, 1999). Hooijer (1972b) alludes to a further collecting trip made by Verhoeven in 1966, but no follow-up reports or other published sources indicate this trip took place, and it’s likely this date represents a typological error in Hooijer’s publication. Following Hooijer’s visit, there appears to have been a hiatus in examination of stegodon sites from Timor until 1993, when Rhys Jones and several other Australian and Indonesian archaeologists visited the fossil-bearing deposits near Atambua. Although a report of the full trip was never formally published by the participants, Jones mentioned it in a book chapter (Jones & Spriggs, 2002), and its results were also briefly reported by O’Connor (2002) on the basis Jones’s field notes and a public lecture. In 1998 Bednarik also revisited Verhoeven’s stegodon localities (Bednarik, 2000), although it seems that he was unaware of Jones et al.’s trip five years previously (O’Connor, 2002). Both sets of researchers were able to successfully relocate the fossil-bearing stratigraphic layer reported by Verhoeven (1964) and the source of the vast majority of stegodon fossils for Timor. In the case of Jones et al.’s fieldwork, geological samples, shells, and at least one stegodon specimen were collected in primary depositional context from the area around Atambua, as well as other material from seven rockshelters from the southern parts of the island (R Jones, 1993, unpublished data; Jones & Spriggs, 2002). Jones found no stone tools in the fossil-bearing stegodon deposits (Jones & Spriggs, 2002; O’Connor, 2002) and concluded that the deposits were likely Early to Middle Pleistocene in age and not associated with humans (Jones & Spriggs, 2002). Because of this lack of primary association between extinct fauna and archaeology, which was the focus of that investigation, no follow-up work on the material collected by Jones and colleagues was undertaken (O’Connor, 2002). However, the material was passed on to one of the authors (SOC), who had in 2001 renewed archaeological research in East Timor. One of the goals of the new research program is examining what effects the first human colonisers had on Timor’s endemic faunas. This research program has also so far produced the earliest dated archaeological material for Timor (O’Connor, 2007). In this context, the possibility that there was a temporal overlap between humans and stegodons requires renewed evaluation, and thus the stegodon specimen collected by Jones et al. was subjected to direct U-series dating.

## Geographic and Geological Setting

Stegodon fossils of Timor were recovered from the Ainaro Gravels, near the village of Atambua (Fig. 1). The Ainaro Gravels are a series of Pleistocene to Holocene-aged river terrace deposits consisting of lenticular beds of generally loosely consolidated boulders, pebbles, sands and silts (Audley-Charles, 1968). Strong cross-bedding is commonly observed, with occasional more lithified conglomerates formed from calcite or lateritic cementing. They outcrop mostly in river channels and valleys throughout Timor, and can reach depths well in excess of 30 m (Fig. 1). Verhoeven (1964) reported finding stegodon fossils from three sites in the Ainaro Gravels. The first was from a dry riverbed around Haleulun, situated in the hills east of Atambua, to which no site name was attached. The second sets of finds were from landslide deposits about 500 m east of Haleulun, an area named Fatukleos, and given the site name Wéaiwé. The third site was described as Fulan Monu, close to Wéaiwé, and located 5 km east of Atambua. Verhoeven describes the stegodon-bearing beds as well rounded, sandy-pebbly conglomerates unconformably overlying fossiliferous marine clay beds. The stegodon specimen examined here was recovered from the site of Wéaiwé by Jones et al. (R Jones, 1993, unpublished data).

## Materials and Methods

### Specimen description and taxonomy

**Figure 2:**
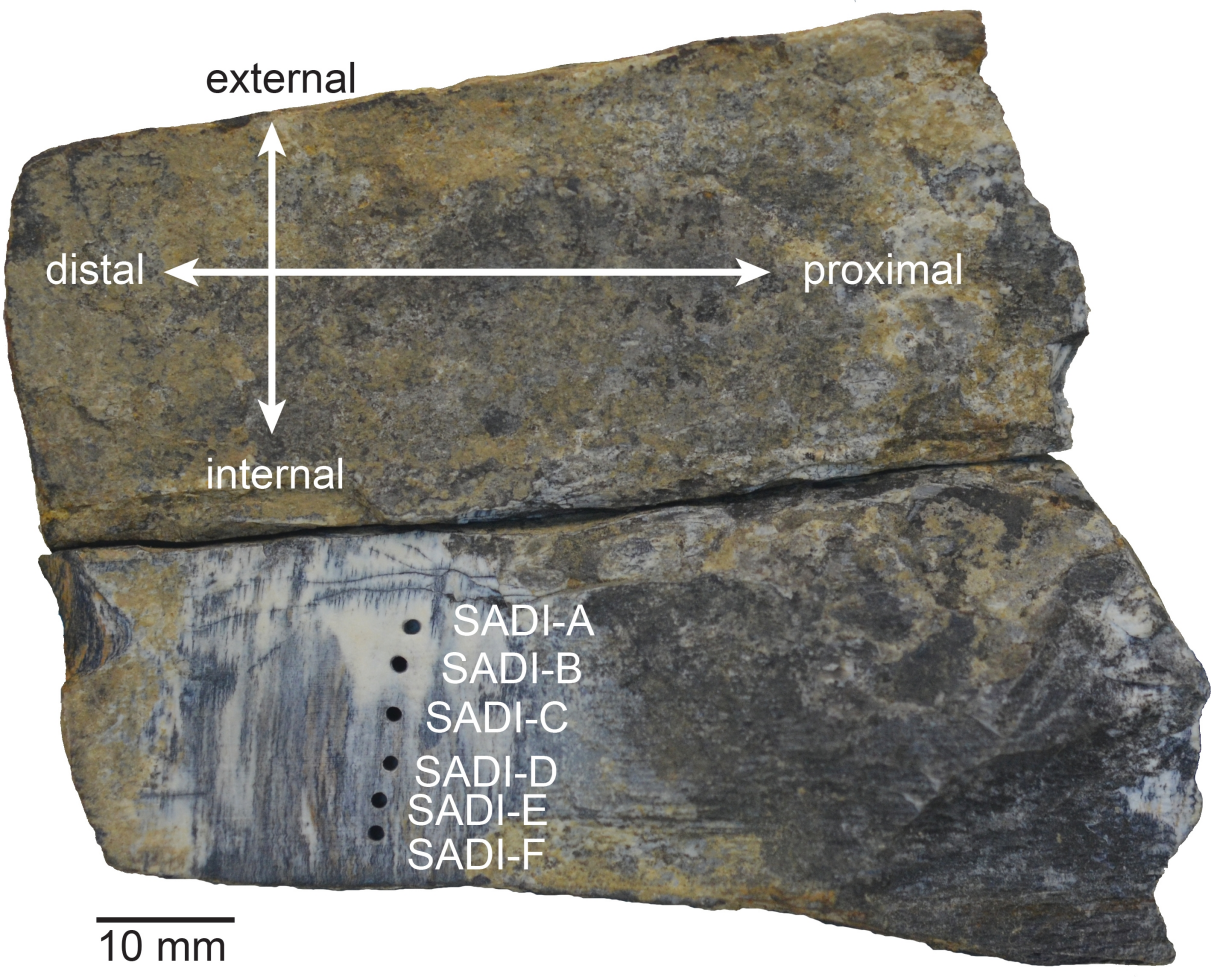
Specimen. Internal view of the refitted stegodon tooth fragments drilled for the uranium series analysis.

The stegodon specimen collected by Jones et al. consists of a partial tusk fragment broken into two pieces along its length (Fig. 2). The major breaks are pre- and syndepositional and the fragments exhibit very slight rounding suggestive of minor fluvial transport. Additional concentric weathering and flaking has resulted in some loss of material from the outer surface of the tooth. The complete reassembled fragment measures 75 mm along the long axis, with a maximum (proximal) cross-sectional diameter of 61.5 mm and a minimal (distal) diameter of 45 mm.

Stegodon material from Timor was first described by Sartono (1969), where on the basis of an incomplete molar he erected the species *Stegodon timorensis*. Sartono (1969) commented on its small size and resemblance to *S. mindanensis*. Hooijer (1969) also remarked on the smallness of *S. timorensis*, which he considered of comparable size to the small species of stegodon recovered from Flores (specifically, Tangi Talo, and eventually described as *S. sondaari* by Van den Bergh (1999)). In fact, the Timorese stegodon is only slightly larger than *S. sondaari* (Van den Bergh, 1999), making it one of the smallest stegodon species known. Hooijer (1969) and Hooijer (1972a) also described Timorese stegodon specimens from a species bigger than *S. timorensis*, although much rarer judging by the number of specimens recovered, including an upper deciduous molar from Sadilaun, molars from Klobor Makerek and Fatubesi, and molar fragments from Biaborlau and Fulan Monu. He ascribed these specimens to *S. trigonocephalus florensis*. This subspecies was elevated to full species by Van den Bergh (1999), although he specifically omitted the Timorese material from the species hypodigm, and the taxonomic status of the large Timorese stegodon thus remains uncertain. The larger stegodon from Timor is herein referred to as *S. ‘trigonocephalus*.’

Hooijer (1972a) and Hooijer (1972b) described several tusk fragments from Timor, none of which he noted where of very large size. They ranged in diameter from 75 to 31 mm, well within the dimensions of the specimen examined herein. Thus, while the material preserved is not diagnostic at the species level, on the basis of its size, locality, and the relative abundance of *S. timorensis* fossils with respect to *S. ‘trigonocephalus*,’ we consider it likely that the specimen dated here belongs to *Stegodon* sp. cf. *S. timorensis*.

### U-Th dating

U-Th dating is based on the premise that living vertebrate tissues contain little to no U; however, during post-mortem diagenesis, bone and teeth can take up U from the burial environment by apatites that scavenge U, but exclude Th (Pike, Hedges & Van Calsteren, 2002). The U-Th age is then calculated by determining the amount of ^230^Th produced from the radioactive decay of ^238^U (via the intermediate isotope ^234^U). Thus, because U is emplaced post-mortem, direct U-Th dating of fossil vertebrate material typically reflects a minimum geological age for the organism at the time of death. In ideal situations, U is taken up rapidly following burial, thus conforming to an early U-uptake model (e.g., Pike, Hedges & Van Calsteren, 2002). In such circumstances, the ^230^Th age will be close to the true age. Alternatively, if U is taken up slowly or is delayed for some time after initial burial, the resulting age can still represent a reliable minimum age, but may more significantly underestimate the ‘true’ age of the vertebrate tissue. It is important to note that bones and teeth are commonly open systems for U following uptake, thus, U may be subsequently leached from the system. In such situations, U-Th dating will typically lead to age overestimation (Grün et al., 2008). In reality, U uptake is vastly complex and multiple different ages can be generated from even a single bone or tooth (Price et al., 2013; Grün et al., 2014). However, it is possible to model ^230^Th age and U concentrations through targeted materials for dating in order to assess the most likely model for U uptake or loss and reliability of the ages (Pike, Hedges & Van Calsteren, 2002; Eggins et al., 2005; Sambridge, Grün & Eggins, 2012; Price et al., 2013).

We obtained six U-Th dates for the Timor stegodon. Samples were drilled sequentially from the inside to the outer margin along a broken cross-section of the tusk (Fig. 2). The drill bits used were only ca. 1 mm diameter, and resulting sample powders weighed on average ca. 1.5 mg each. The sampling approach followed Price et al. (2013) because it allows for tight spatial control of individual samples through the tooth. Laser ablation can also generate spatially-controlled profiles through teeth, but typically means that a given tooth must be cut and slabbed down to size in order to fit into a vacuum chamber (Grün et al., 2014), a luxury that we didn’t have for the specimen. The samples were measured on a multi-collector inductively coupled plasma mass spectrometer (MC-ICP-MS) at The University of Queensland. The sampling approach allowed for the construction of ^230^Th age and U-concentration profiles through the tooth, thus enabling us to evaluate the tooth in providing reliable minimum ages. Chemical pre-treatment followed techniques described in Price et al. (2013) and measurement on the MC-ICP-MS followed the procedures outlined in Zhou et al. (2011) and Roff et al. (2013). All errors are reported to 2*σ*.

## Results and Interpretation

**Figure 3:**
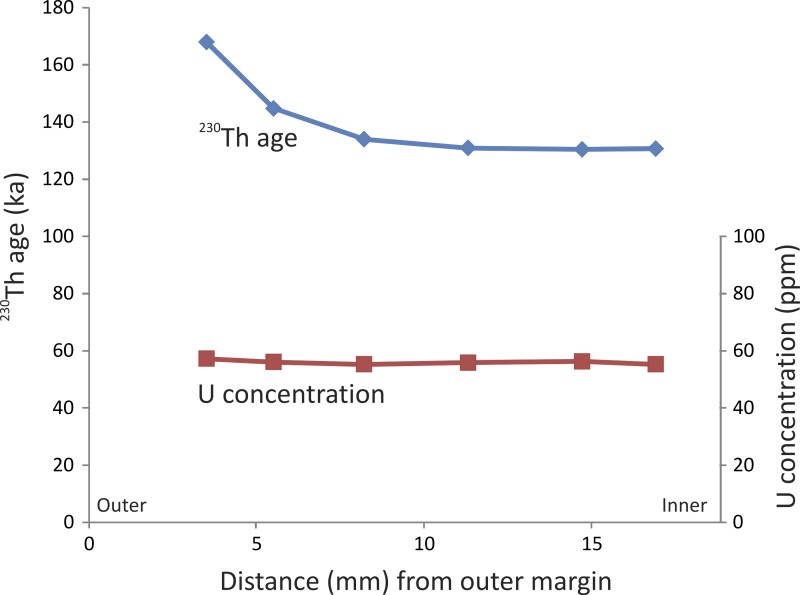
Profile through tooth. ^230^Th age and U concertation profiles through the Timor stegodon tusk (note: error bars are very small and cannot be discerned at the scale plotted. See (Table 1) for full U-series isotopic and concentration data).

The six ^230^Th ages from the stegodon tusk range from ca. 130 to 168 ka (Table 1). The samples are typically characterised by high concentrations of U (Table 1), regardless of distance from the external margin (Fig. 3). The samples are very clean with respect to detrital ^232^Th, thus the ^230^Th ages have very high precision of ca. 0.5%.

**Table 1:** U-series data. U-series isotopic and concentration data for the Timor stegodon tusk. All ages reported to 2*σ* error.

Sample name	U (ppm)	^232^Th (ppb)	(^230^Th∕^232^Th)	(^230^Th∕^238^U)	(^234^U∕^238^U)	Uncorr. age (ka)	Corr. age (ka)	Corr. initial (^234^U∕^238^U)
SADI-A	55.25 ± 0.02	2.90 ± 0.06	71432	1.2359 ± 0.0041	1.6603 ± 0.0018	130.7 ± 0.8	130.7 ± 0.8	1.9547 ± 0.0028
SADI-B	56.28 ± 0.05	5.10 ± 0.07	40657	1.2143 ± 0.0040	1.6353 ± 0.0017	130.4 ± 0.8	130.4 ± 0.8	1.9179 ± 0.0026
SADI-C	55.82 ± 0.04	5.58 ± 0.06	36418	1.2003 ± 0.0030	1.6148 ± 0.0014	130.9 ± 0.6	130.9 ± 0.6	1.8896 ± 0.0021
SADI-D	55.25 ± 0.03	1.00 ± 0.06	199456	1.1938 ± 0.0026	1.5883 ± 0.0016	133.9 ± 0.6	133.9 ± 0.6	1.8584 ± 0.0021
SADI-E	56.09 ± 0.03	1.16 ± 0.08	179401	1.2238 ± 0.0030	1.5633 ± 0.0015	144.7 ± 0.7	144.7 ± 0.7	1.8475 ± 0.0022
SADI-F	57.22 ± 0.03	1.79 ± 0.06	127402	1.3164 ± 0.0038	1.5602 ± 0.0011	168.0 ± 1.1	168.0 ± 1.1	1.9000 ± 0.0028

**Notes.**

Ratios in parentheses are activity ratios calculated from the atomic ratios, but normalized to measured values of secular-equilibrium HU-1 standard following the method of Ludwig et al. (1992). Errors are at 2*σ* level. ^230^Th ages are calculated using Isoplot EX 3.0 (Ludwig, 2003) with decay constants *λ*_238_ = 1.551 × 10^−10^ yr^−1^ (for ^238^U), *λ*_234_ = 2.826 × 10^−6^ yr^−1^ (for ^234^U) and *λ*_230_ = 9.158 × 10^−6^ yr^−1^ (for ^230^Th), respectively (after Cheng et al., 2000). 2*σ* errors in the uncorrected (uncorr.) ages were propagated directly from the uncertainties in the (^230^Th∕^238^U) and (^234^U∕^238^U). The corrected (corr.) ^230^Th age was calculated assuming the non-radiogenic U-Th component with bulk-earth ^232^Th∕^238^U atomic ratio of 3.8 ± 1.9 (^230^Th, ^234^U and ^238^U are assumed to be in secular equilibrium). Such corrections generally have little impact on the ages of the tooth samples.

It is important to note that the ages are oldest on the external margin and become progressively younger closer to the middle portion of the tusk (Fig. 3). Such variation in a ^230^Th age profile through a single tooth is not necessarily unexpected (Price et al., 2013). Although we did not sample across the entire diameter of the broken tusk, the resulting ^230^Th age profile clearly resembles a half ∪-shaped pattern suggesting relatively slow, but continuous, U uptake under constant conditions. Alternatively, loss of U could also potentially lead to anomalously older ages on the external margin relative to the interior samples. However, if that was the case, then we might expect to see evidence for U loss in such samples; for example, samples on the outer margin may have significantly lower U-concentrations than those in the middle of the tusk, resulting in a ∩-shaped profile. However, the U-concentration profile through the stegodon tusk does not show marked evidence of U loss on the margin. Rather, U-concentration is fairly uniform through the tooth (Fig. 3). It is possible though that U-concentration was initially higher on the outer margin (i.e., much greater than 57 ppm) and that U has been lost from that portion of the tooth resulting in ^230^Th ages markedly older than in the middle. It may simply just be a coincidence that U-concentration on the outer and inner margins is now similar.

Regardless, it is evident that three statistically indistinguishable ages occur in the middle of the tooth, centred around 130 ka (SADI-A, SADI-B, and SADI-C). Similarly, corresponding U-concentrations are also virtually identical for those three samples (i.e., U-concentration of 55–56 ppm). Those observations suggests that for at least that part of the tooth, the U-concentration profile is in equilibrium and that the ‘plateau’ age profile suggests early uptake of U following burial (e.g., Pike, Hedges & Van Calsteren, 2002). Although the outer margin of the tooth may have experienced U-loss, the middle portion has a reliable ‘closed system’-like profile, and thus, has yielded reliable minimum ages. We are confident that the Timor stegodon tusk is minimally 130 ka or slightly older, making it at latest Middle Pleistocene in age.

## Discussion

Our dating results allow us to test the hypotheses that humans and proboscideans were coeval on the island, and that humans had a role in their extinction. Like all hypotheses examining the role of humans in Quaternary extinctions, this question revolves around two events, namely the date of extinction of the taxa in question, and the date of first arrival of humans (Price et al., 2011; Price et al., 2015; although obviously the temporal correlation of humans and extinct fauna does not automatically indicate causation). While our dating results do not provide an answer on the first event, it does serve to better temporally constrain local stegodon. As our new dates represent the first and so far only directly dated stegodon samples from Timor, it constitutes a terminus ante/post quem for this taxon. Obviously, additional dated samples from other localities would be required to reliably estimate the timing of stegodon extinction on Timor. While Glover (1986) pointed out that the Ainaro Gravels in East Timor had potential for producing more stegodon bearing deposits, unfortunately, and despite our extensive survey in areas where this formation is exposed, no stegodon-bearing deposits have been identified other than those previously reported in the Atambua area.

Determining the timing of arrival of modern humans in Timor, and in fact in East Nusa Tenggara altogether, is hampered by a lack of archaeological evidence throughout the region coupled with an almost complete absence of pre-human palaeontological records (O’Connor & Aplin, 2007; O’Connor et al., 2010; Kealy, Louys & O’Connor, 2015). The island with the oldest archaeological sequence in the region is Flores. A currently unknown, tool-making hominin arrived on Flores ca. 900 ka (Brumm et al., 2010). The idea that Pleistocene hominins other than *Homo sapiens* made it significantly further eastwards than Flores is no longer seriously entertained due to a complete lack of any evidence suggesting otherwise (Allen, 1991; Jones & Spriggs, 2002), and is considered here highly unlikely although not presently possible to refute. Evidence for occupation of Flores by *H. floresiensis* dates from ca. 95 ka to 17 ka (Westaway et al., 2009c). Modern humans did not arrive on Flores until some point shortly after 12 ka (Morwood & Van Oosterzee, 2007); however, as O’Connor et al. (2010) point out, a post-12 ka date for the arrival of *Homo sapiens* on Flores is anomalous considering other archaeological evidence from the region suggesting modern humans in Timor at least 42 ka, and on nearby Alor by at least 20 ka (Samper Carro et al., 2015).

Evidence for the presence of Pleistocene *Homo sapiens* in Wallacea is restricted to only a few islands, but by far the best archaeological records come from Timor. Early claims of direct association between the stone tools recovered in the vicinity of Atambua and stegodons (Glover & Glover, 1970; Maringer & Verhoeven, 1970; Maringer & Verschuuren, 1981) have since been treated with a high degree of scepticism (e.g., Allen, 1991; Jones & Spriggs, 2002; O’Connor, 2002), and Timor’s archaeological record has improved considerably since these claims were made. There are now several sites (Lene Hara, Jerimalai, Matju Kuru) that have yielded both diverse faunal and lithic assemblages dating back more than 40 ka (O’Connor, Spriggs & Veth, 2002; O’Connor, Ono & Clarkson, 2011; O’Connor, Robertson & Aplin, 2014). However, no assemblages contain stegodons. The only land mammals excavated from the earliest archaeological levels are giant murids (*Coryphomys* spp. and three other undescribed genera), and up to five smaller-bodied, but presently undescribed endemic rodents (Aplin & Helgen, 2010).

While none of these East Timorese sites have yet produced pre-human deposits, it’s likely that the earliest human colonisation of the island occurred shortly before the earliest archaeological deposits, i.e., sometime around 50–60 ka. This is because the earliest arrival dates of humans in Australia, long accepted to have occurred by at least 45 ka (Allen & O’Connell, 2014)—and with initial occupation reported to around 60 ka from northern Australia (Madjedbebe; Clarkson et al., 2015)—would have necessitated maritime travel through Wallacea and almost certainly Timor (Birdsell, 1977; Kealy, Louys & O’Connor, 2015). Other sites in Wallacea producing Pleistocene dates support this timing (Fig. 1). Aubert et al. (2014) dated rock art unambiguously associated with modern human behaviour on Sulawesi to around 40 ka (Leang Timpuseng), and archaeological sites from the islands of Salibabu and Gebe have yielded dates of around 30 ka (Ono, Soegondho & Yoneda, 2009; Bellwood et al., 1998). These dates are congruent with the earliest dated modern humans from southern China (>80 ka; Liu et al., 2015) and continental Southeast Asia (63–46 ka; Demeter et al., 2012), suggesting that modern human dispersal through Southeast Asia was a relatively quick phenomenon occurring on a timescale of a few tens of thousands of years.

Our date of ca. 130 ka for stegodon on Timor, in conjunction with the earliest archaeological dates from East Timor at 42 ka (O’Connor, Ono & Clarkson, 2011), suggest a gap of at least 80 ka between occurrences. On balance, we consider it highly unlikely that any hominins and stegodons overlapped on Timor, and thus the extinction of stegodon on the island would have been due to other factors impacting on the population prior to the arrival of modern humans. Due to a dearth of palaeoenvironmental records from Timor, it is difficult to properly evaluate the scale of ecological changes occurring during the Middle to Late Pleistocene on the island. Regional palaeoenvironmental data sheds some light on the possible climatic and environmental factors that acted on a regional scale. The late Quaternary record from Liang Bua, Flores indicates that local environmental conditions fluctuated between closed woodland vegetation with high but unstable rainfall to periods of decreased humidity and concomitant shifts to C4 vegetation (Westaway et al., 2009a; Westaway et al., 2009b). However, the faunal record from Liang Bua demonstrates that stegodon survived these fluctuations without extinction. In fact the distribution of faunal material in that cave follows the intensity of human occupation rather than responses to differing environmental conditions (Westaway et al., 2009b). Extinction of the Late Pleistocene stegodon on Flores is hypothesised to have occurred as a result of a volcanic eruption (Van den Bergh et al., 2009) as stegodon material is found below a volcanic layer dated to ca. 17 ka, but is entirely absent above it. A much earlier extinction of stegodon is also recorded on Flores, when the dwarf stegodon *Stegodon sondaari* was replaced by the slightly larger, but still small-bodied *S. florensis* in a turnover occurring between the Early Pleistocene Tangi Talo deposits and those of the slightly later Mata Menge, Boa Lesa and Kobatuwa localities (Meijer et al., 2010; Dennell et al., 2014). What caused this turnover is uncertain, and it isn’t completely clear whether this was a single (e.g., a volcanic eruption) or staggered event (Brumm et al., 2010). Ecological replacement of *S. sondaari* by *S. florensis* also seems plausible.

Middle to middle-Late Pleistocene environmental data comes from further afield than Flores, namely Java to the west. There, Middle Pleistocene environments have been reconstructed as mosaic of diverse evergreen forests, shrubby woodland, and extensive grasslands similar to modern day East Africa or India (Medway, 1972; De Vos et al., 1994; Van den Bergh, De Vos & Sondaar, 2001; Bouteaux, 2005; Louys & Meijaard, 2010). By around 135 ka, around the date of the Timor stegodon, annual precipitation on Java is estimated to have been much lower than today (Van der Kaars & Dam, 1995; Van der Kaars & Dam, 1997). Nevertheless, by around 126–81 ka, very warm and humid conditions returned, as did the rainforests (Van der Kaars & Dam, 1995; Van der Kaars & Dam, 1997; Westaway et al., 2007). Later Pleistocene environmental changes on Java continued to fluctuate between drier, more open glacial periods (e.g., MIS 2) and wetter, more humid interglacials (Louys & Meijaard, 2010).

It seems likely that vegetation communities on Timor would have fluctuated in a similar fashion to Java and Flores throughout the Middle to Late Pleistocene, although Flores’s palaeontological history suggests such fluctuations would not have caused the extinction of stegodon on a large oceanic island such as Timor. Nevertheless, island ecosystems, by the nature of their more spatially and biologically limited resources, relative isolation, and less complex ecological communities, exert significant ecological pressure on populations of large mammals (Palombo, 2007; Lomolino et al., 2012; Lomolino et al., 2013), and the dwarfed size of stegodons on Timor almost certainly attests to such pressures. While the smaller size of the Timorese stegodons may have been the result paedogenesis, associated with higher reproductive rates and maintaining genetic diversity (c.f. Palombo, 2007); equally Timor, although a relatively large island, is isolated, ecologically simple (for its size), and lacks any endemic mammalian carnivores as well as similar sized herbivores: factors considered important in explaining the size reduction of large mammals on islands, including proboscideans (Lomolino et al., 2012; Lomolino et al., 2013).

While speculative, we offer the following possible extinction scenario for Timor’s stegodons. Insular populations of this species may have been able to survive repeated instances of genetic bottlenecking resulting from fluctuating environmental conditions, but with an overall downward trajectory for their survival as a species. Given prolonged isolation, or speciation of insular populations progressing to the point where existing residents can’t hybridise with new migrants, such a trajectory may play out on the scale of tens of thousands of years, leading to the eventual global extinction of endemic island species without an obvious or (geologically) discernible trigger.

## Additional Information and Declarations

### Data Availability

The following information was supplied regarding data availability: The raw data is contained in the manuscript.
